# Vanadate from Air Pollutant Inhibits Hrs-Dependent Endosome Fusion and Augments Responsiveness to Toll-Like Receptors

**DOI:** 10.1371/journal.pone.0099287

**Published:** 2014-06-05

**Authors:** Mojca Zelnikar, Mojca Benčina, Roman Jerala, Mateja Manček-Keber

**Affiliations:** 1 National Institute of Chemistry, Laboratory of Biotechnology, Ljubljana, Slovenia; 2 EN→FIST Centre of Excellence, Ljubljana, Slovenia; IISER-TVM, India

## Abstract

There is a well-established association between exposure to air pollutants and pulmonary injuries. For example, metals found in ROFA (residual oil fly ash) increase susceptibility of mice as well as humans to microbial infections. In our research, we have found that vanadate substantially increased the response of several Toll-like receptors (TLRs) to stimulation with their ligands. Although vanadate caused generation of reactive oxygen species (ROS), the addition of ROS scavenger N-acetyl cysteine (NAC) had no effect on augmented lipopolysaccharide (LPS) stimulation. We further showed that vanadate inhibits endosome fusion. This effect was determined by measuring the size of endosomes, NF-κB activity and TLR4 degradation in Hrs (hepatocyte growth factor-regulated tyrosine kinase substrate) overexpressed cells. Moreover, we identified the role of Hrs phosphorylation in these processes. Based on our findings, we can conclude that vanadate potentiates TLR4 activity by increasing Hrs phosphorylation status, reducing the size of Hrs/TLR4-positive endosomes and impacting TLR4 degradation, thus contributing to the detrimental effects of air pollutants on human health.

## Introduction

All living organisms are constantly exposed to an external environment filled with microorganisms and air pollutant particles. Epidemiological research has demonstrated a correlation between increased morbidity and mortality of humans and higher concentrations of pollutant particles in ambient air [1]. Fine particles with a diameter less than 2.5 µm are more closely related to adverse effects on human health than are bigger particles [2]; adverse effects are often the result of partial oxidation of carbonaceous material. Analysis of residual oil fly ash (ROFA) produced by oil combustion has shown that fly ash is a chemically complex mixture of sulphates, silicates, carbon and nitrogen components and various oil contaminants. High concentrations of water soluble metal ions, such as iron, vanadium and nickel are present in ROFA as well [3].

ROFA and particles found in the exhaust dust of diesel fuel (DEP) as well as gases (ozone, SO_2_, NO_2_) cause inflammation and damage to the airway [4], [5], [6], [7]. Mechanisms of airway damage after exposure to airborne pollutant particles are not fully elucidated, as they are likely to be diverse. It was proposed that water soluble metal ions (vanadium (V), nickel (II) and iron (III) ions) in ROFA stimulate production of reactive oxygen species (ROS) [8], [9], [10]. Short-term exposure to particles increases ROS levels in the heart and lungs of rats [11], and pretreatment with the antioxidant N-acetyl cysteine (NAC) inhibits inflammation [12]. Formation of ROS after ROFA stimulation up-regulates NF-κB and AP-1 signaling [13], cytokine and iNOS expression [14], and also causes lipid peroxidation [15] and genotoxic effects [9], [16]. Susceptibility of mice as well as humans to bacterial infections increases upon exposure to ROFA in correlation to its metal content [17], [18].

The role of Toll-like receptors (TLRs) as innate immunity receptors, which recognize microbial substances as well as endogenous danger signals, has already been well established. Activation of TLRs is initiated through ligand-induced receptor dimerization. Subsequently, MyD88 or TRIF adaptor proteins are recruited to TIR domains of TLRs, leading to the assembly of complex intracellular signaling, which in turn leads to the activation of a phosphorylation cascade and synthesis of proinflammatory cytokines and/or type I IFN expression (reviewed in [19]). Termination of TLR signaling is an important step in regulating the intensity of inflammation. It was shown that Hrs protein (hepatocyte growth factor-regulated tyrosine kinase substrate) sorts ubiquitinated receptors and other proteins to multivesicular bodies (MVB), thereby terminating the signaling and promoting lysosomal degradation of receptors [20]. After lipopolysaccharide (LPS) stimulation, TLR4 is ubiquitinated and binds to Hrs on endosomes; desensitization to LPS proceeds [21].

C3H/HeJ mice containing a dominant negative mutation in TLR4 are partially resistant to ROFA- [22] and DEP-induced [23] injury. Additionally, prestimulation with ROFA decreased tracheal antimicrobial peptide (TAP) induction in response to lipopolysaccharide (LPS), a TLR4 ligand [24]. This activity has been attributed to vanadium, but not to nickel or iron.

In this report we show that vanadate increased the sensitivity of cells to TLR ligands. Several reports showed vanadate acts through ROS production. We also detected increased ROS production in cells treated with vanadate and LPS. However, antioxidant NAC had no effect on the synergistic contribution of vanadate on LPS signaling when NF-κB activity was measured. Vanadate treatment prolonged LPS-induced MAPK phosphorylation, attributable to the ability of vanadate to inhibit phosphatases. Vanadate-dependent phosphorylation of Hrs inhibited endosome fusion, decreased TLR4 degradation, and thus increased LPS signaling. As vanadate had the same effect on signaling through other tested TLR receptors, the mechanism was not found to be specific for TLR4 and this can explain the increased susceptibility of humans exposed to ROFA to microbial infections.

## Materials and Methods

### Reagents

N-acetyl-L-cystein (NAC), protease inhibitors, and sodium orthovanadate (Na_3_VO_4_) were from Sigma. S-LPS from *Salmonella enterica* serotype *abortus equi* was a gift from Dr. Brandenburg (Borstel, Germany). Phosphatase inhibitors, set III, was from Merck and 2′,7′-dichlorodihydrofluorescein diacetate (H_2_DCFDA), flagellin, poly(I:C), and Pam_2_CSK_4_ were from Invitrogen. Goat anti-mouse was from Santa Cruz Biotechnology, anti-tubulin from NEB, anti-GFP from Abcam, anti-Myc from Sigma, and anti P-p44/42 MAP kinase was from Cell Signaling Technology. LPS was labeled with Cy5 bifunctional reactive dye (part of labeling kit PA35000; GE Healthcare).

### Cell Cultures

HEK293 human embryonic kidney cell line, A549 human cancer alveolar basal epithelial cells and RAW264.7 mouse macrophages (all from ATCC) were used. HEK293, A549 cells were cultured in DMEM with glutamax (Invitrogen) supplemented with 10% FBS (Invitrogen). RAW264.7 cells were cultured in RPMI 1640 with glutamax supplemented with 10% FBS. Cells were grown in tissue culture flasks at 37°C and 5% CO_2_.

### Dual Luciferase Assay

HEK293 cells were transiently transfected with TLR4/MD-2/CD14, TLR5, TLR3, or Hrs expression plasmids, as well as NF-κB or IRF3 luciferase and Renilla luciferase reporter plasmids. Cells were stimulated with 100 ng/ml LPS, 50 ng/ml flagellin or 10 µg/ml poly(I:C), with or without Na_3_VO_4_ (500 µM) prestimulation. To inhibit ROS activity, cells were preincubated with 5 mM NAC. Cells were lysed and analyzed for reporter gene activities using a dual luciferase reporter assay system on Orion luminometer (Bertholod). Data from luciferase activity were normalized using Renilla luciferase readings.

### Elisa

96-well plate was seeded with A549 cells, prestimulated with 100 or 500 µM Na_3_VO_4_ and stimulated with LPS (1 µg/ml), Pam_2_CSK_4_ (200 ng/ml) or poly(I:C) (50 µg/ml). Human IL-6 was determined in supernatants (eBioscience).

### Western Blot

RAW264.7 cells were stimulated with Na_3_VO_4_ (2 mM), Na_3_VO_4_ and LPS (100 ng/ml) or LPS alone for different times (0, 5, 10, 15, 20, 40, 60 and 120 min). Cells were lysed and 30 µg of proteins were loaded to SDS-PAGE. Phosphorylated p44/42 (P-p44/42) was detected and anti-tubulin Abs were used for normalization.

HEK293 cells, stably expressing TLR-CFP/MD-2 and transfected with Hrs were treated with 80 µg/ml cycloheximide, which was added 30 minutes before stimulation and cells were stimulated with LPS (100 ng/ml) or Na_3_VO_4_ (500 µM) and LPS. After 3 hours, cells were lysed and 30 µg of proteins were loaded to SDS-PAGE. Anti-GFP Abs were used to detect TLR4-CFP and anti-tubulin Abs for normalization. Band intensities were analysed using GeneTools (SynGene).

### Confocal Microscopy

HEK293 cells stably expressing TLR4-CFP/MD-2 were transiently transfected with CD14 and Hrs-YFP or HrsY329/334F-EGFP. Cells were stimulated with 500 µM Na_3_VO_4_ and/or 2 µg/ml LPS-Cy5. Cells were fixed with 4% paraformaldehide. Cells were observed using a Leica TSC SP5 confocal microscope equipped with an HCX plan apo 963 (NA 1.4) oil immersion objective. Excitation/emission conditions in imaging for CFP, EGFP, YFP and Cy5 were 405 nm/460–500 nm, 515 nm/525–560 nm and 633 nm/650–700 nm, respectively. For acquisition and image processing, Leica LAS AF software was used. A minimum of 10 images were taken and analyzed for endosome sizes using image analysis software, ImageJ.

### Flow Cytometry

On a 6-well plate, A549 or HEK293 cells were seeded and incubated overnight. The next day, the A549 cells were dyed with 0.2 µM H_2_DCFDA, pretreated for 10 min with Na_3_VO_4_ (100 or 500 µM) and then stimulated for 4 h with 1 µg/ml LPS or only treated for 4 h with Na_3_VO_4_ (100, 500 or 1000 µM). HEK293 cells were dyed with 0.2 µM H_2_DCFDA, where indicated pretreated for 30 min with 5 mM NAC and then treated for 4 h with Na_3_VO_4_ (500 µM). After stimulation cells were analyzed by flow cytometer analysis, where fluorescent 2′,7′-dichlorofluorescein DCF, produced by ROS was measured at 488 nm excitation and 525 nm emission wavelengths.

### Statistical Analysis

Representative experiments are shown. For the analysis of experimental data, a *t*-test was used. For the analysis of the size of endosomes one way ANOVA analysis with Turkey post analysis was used. *P<0.1; **P<0.05; ***P<0.01; **** *P*<0.005; n.s. - not significant.

## Results

### Vanadate Increased Responsiveness to TLR Ligands, but not through ROS Production

**Figure 1:**
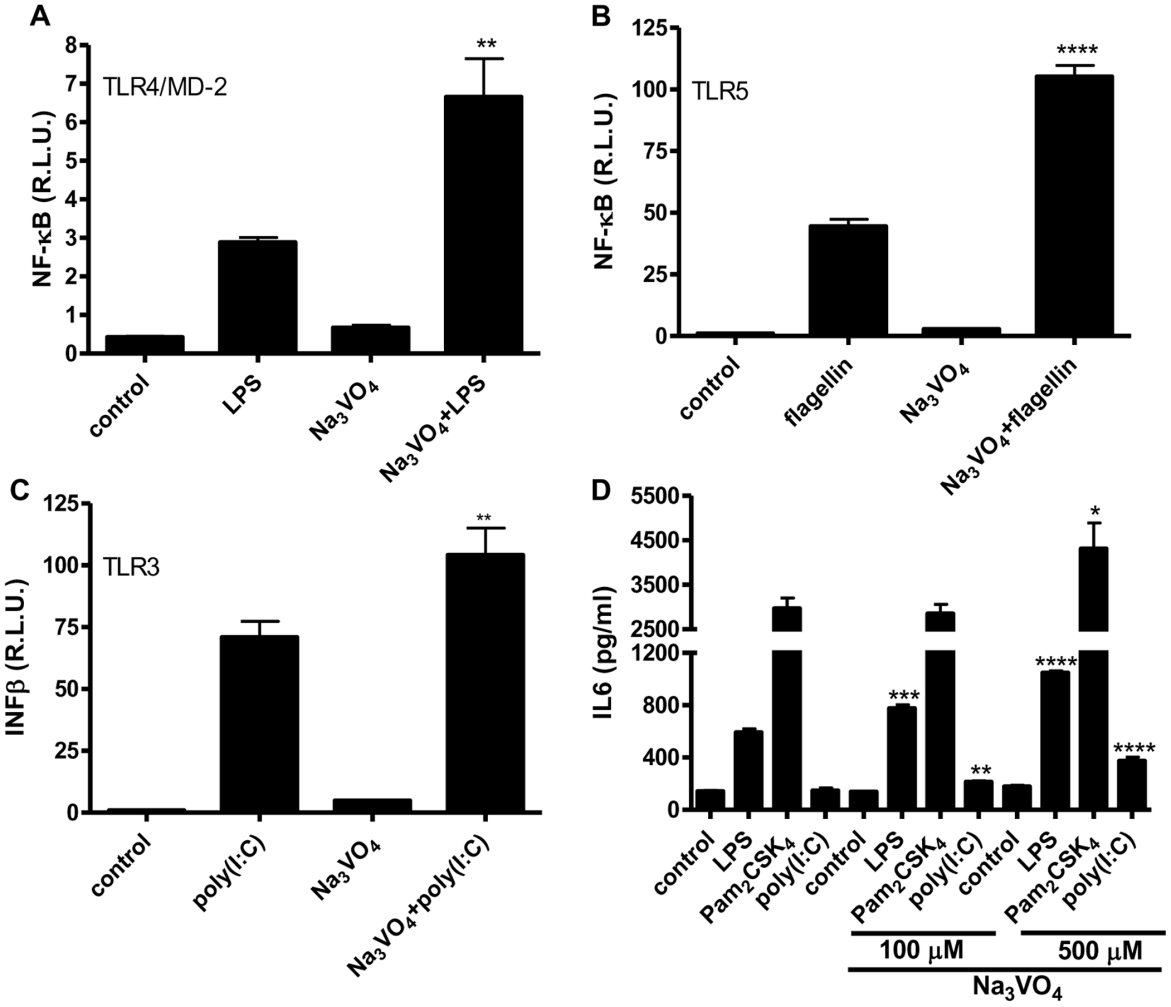
Vanadate increased signaling through TLR receptors. HEK293 cells expressing TLR4/MD-2/CD14 were stimulated for 16 h with LPS (100 ng/ml) **A.**; TLR5 with flagellin (50 ng/ml) **B.**; TLR3 with poly(I:C) (10 µg/ml) **C.** and/or prestimulated for 10 min with Na_3_VO_4_ (500 µM). A dual luciferase test for NF-κB or IFNβ activity was performed. **D.** A549 were stimulated for 16 h with LPS (1 µg/ml), poly(I:C) (50 µg/ml), or Pam_2_CSK_4_ (200 ng/ml) and/or prestimulated for 10 min with Na_3_VO_4_ (500 µM). ELISA was performed for human IL6. Representative examples of three (A–C) and two (D) independent experiments are shown.

Most inflammatory effects of ROFA are attributed to metal ions, particularly vanadium, which is present as a water soluble salt (vanadate). We wanted to investigate the effect of sodium orthovanadate on Toll-like receptors. HEK293 and A549 cells were pretreated with vanadate and stimulated with several TLR ligands. The addition of vanadate increased signaling through surface-expressed TLR2/6, TLR4, TLR5, and through endosomal TLR3 receptors, detected by the increased NF-κB or IFNβ activity (Figures 1A–C) and IL6 production (Figure 1D).

**Figure 2:**
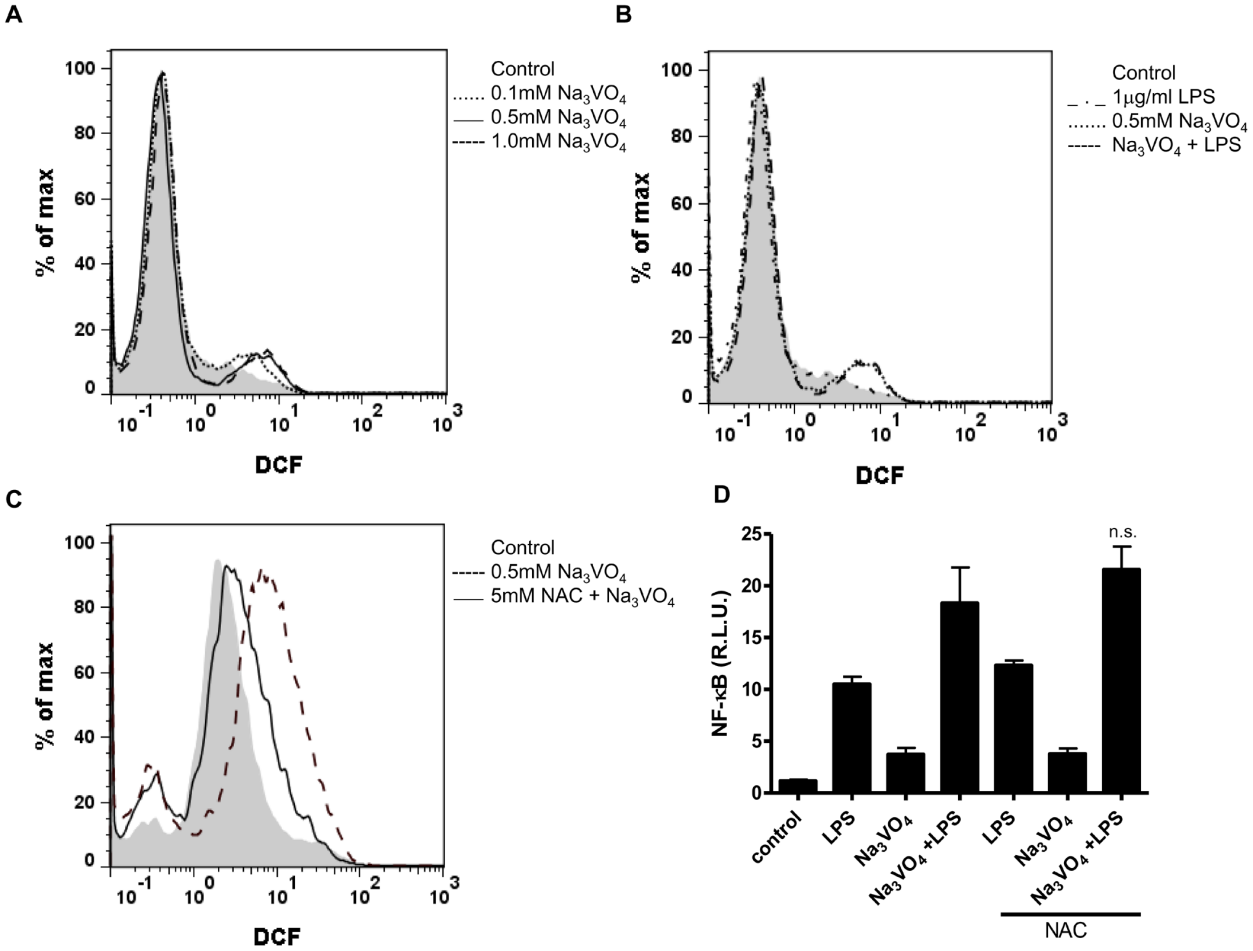
Vanadate-induced generation of ROS, which did not influence NF-κB activity. **A, B.** A549 cells were stained with 0.2 µM H_2_DCFDA for 15 min, (pre)stimulated with Na_3_VO_4_ (0.1, 0.5 or 1.0 mM) and/or LPS (1 µg/ml). Fluorescence of DCF (525 nm) was measured by flow cytometer. **C.** HEK293 cells were stained with 0.2 µM H_2_DCFDA for 15 min, preincubated for 30 min with 5 mM antioxidant NAC and treated with Na_3_VO_4_ (0.1, 0.5 mM) for 4 h. Fluorescence was measured by flow cytometer. **D.** HEK293 expressing TLR4/MD-2/CD14 were preincubated for 30 min with 5 mM NAC and prestimulated for 10 min with Na_3_VO_4_ (500 µM) and/or stimulated for 16 h with LPS (100 ng/ml). A dual luciferase test for NF-κB activity was performed. Representative examples of two (A, B, C) and three (D) independent experiments are shown.

Vanadium has been shown to cause several cell activation effects, probably by influencing different signaling pathways [9], [13]. The addition of vanadate to A549 cells stimulated ROS production in a concentration-dependent manner, as measured by the increase in the fluorescent ROS sensor, DCF (Figure 2A), but only minimaly augmented LPS-induced ROS production (Figure 2B). The addition of vanadate to HEK293 cells had the same effect and the pretreatment with antioxidant NAC decreased vanadate-induced ROS production (Figure 2C). However, the augmented NF-κB response to LPS due to the presence of vanadate remained the same regardless of the NAC present (Figure 2D), demonstrating that vanadate-stimulated ROS production is not responsible for the increased NF-κB responsiveness induced by TLR4 receptor with LPS.

### Vanadate Influenced the Sizes of Endosomes and Compensated for the Hrs Inhibition of LPS Signaling

**Figure 3:**
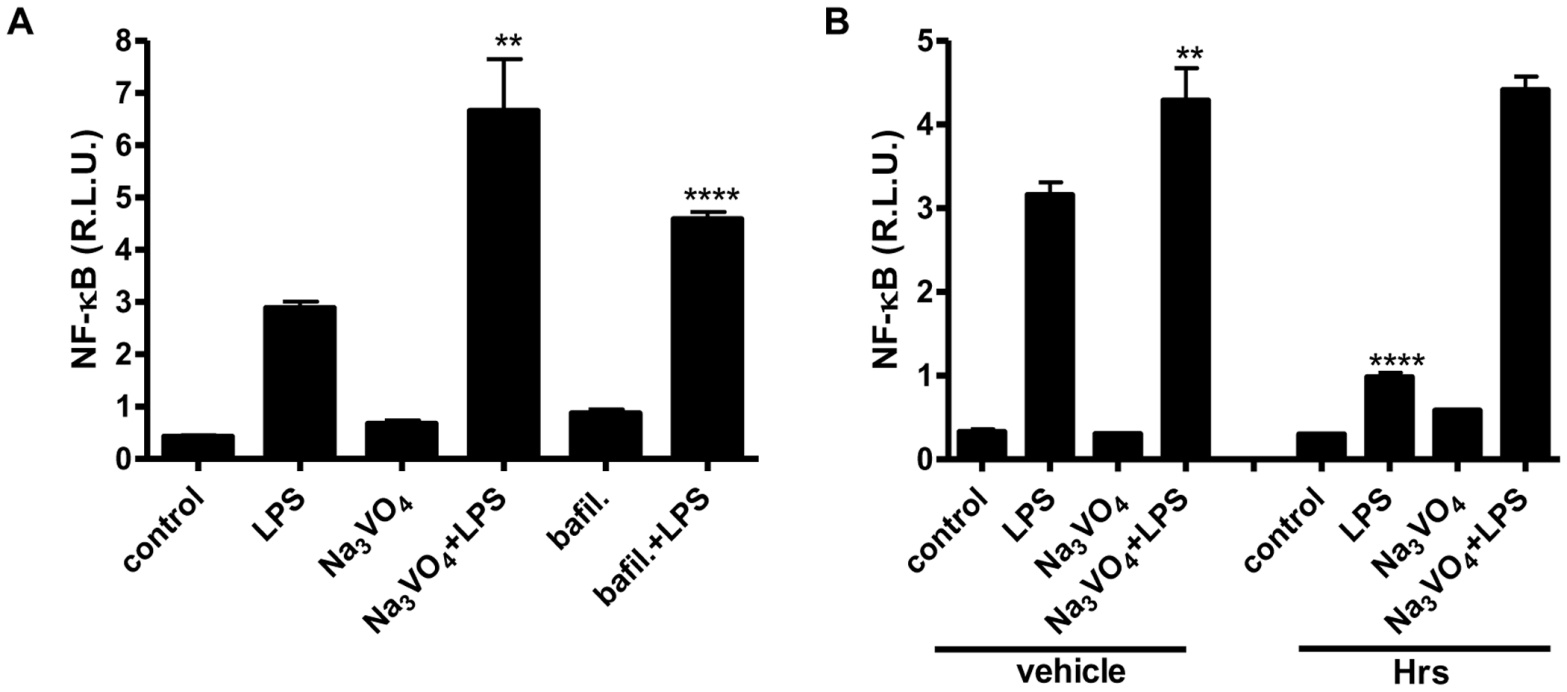
Vanadate reduced the size of endosomes and compensated for the inhibitory effect of Hrs on LPS stimulation. **A.** HEK293 expressing TLR4/MD-2/CD14 were prestimulated for 10 min with Na_3_VO_4_ (500 µM) or bafilomycin (0.15 µM) and/or stimulated for 16 h with LPS (100 ng/ml). **B.** HEK293 expressing TLR4/MD-2/CD14 and/or Hrs were prestimulated for 10 min with Na_3_VO_4_ (500 µM) and/or stimulated for 16 h with LPS (100 ng/ml). A dual luciferase test for NF-κB activity was performed. Representative examples of two (A) and three (B) independent experiments are shown.

HEK293 cells that stably expressed TLR4-CFP/MD-2 were left untreated, or were treated with vanadate. After the addition of vanadate, smaller sized endosomes were observed in comparison to the untreated controlas shown later. The effect of vanadate on cell stimulation was comparable to bafilomycin, an inhibitor of vacuolar H+ ATPase, which inhibits fusion between endo/lysosomes, and thus increases LPS signaling [25] (Figure 3A), suggesting a similar mechanism for vanadate.

As already mentioned, Hrs protein is responsible for sorting ubiquitinated TLR4 to endosomes and for its degradation. This process was confirmed by Hrs siRNA transfection, which increased LPS signaling [21]. We transfected HEK293 cells with TLR4/MD-2/CD14, with and without Hrs, pretreated them with vanadate and stimulated them with LPS. As expected, Hrs overexpression alone decreased LPS signaling (Figure 3B). Suprisingly, the addition of vanadate compensated for the effect of Hrs on LPS signaling (Figure 3B), indicating that vanadate might influence endosome fusion by acting on Hrs protein.

### Vanadate Inhibits Endosome Fusion

**Figure 4:**
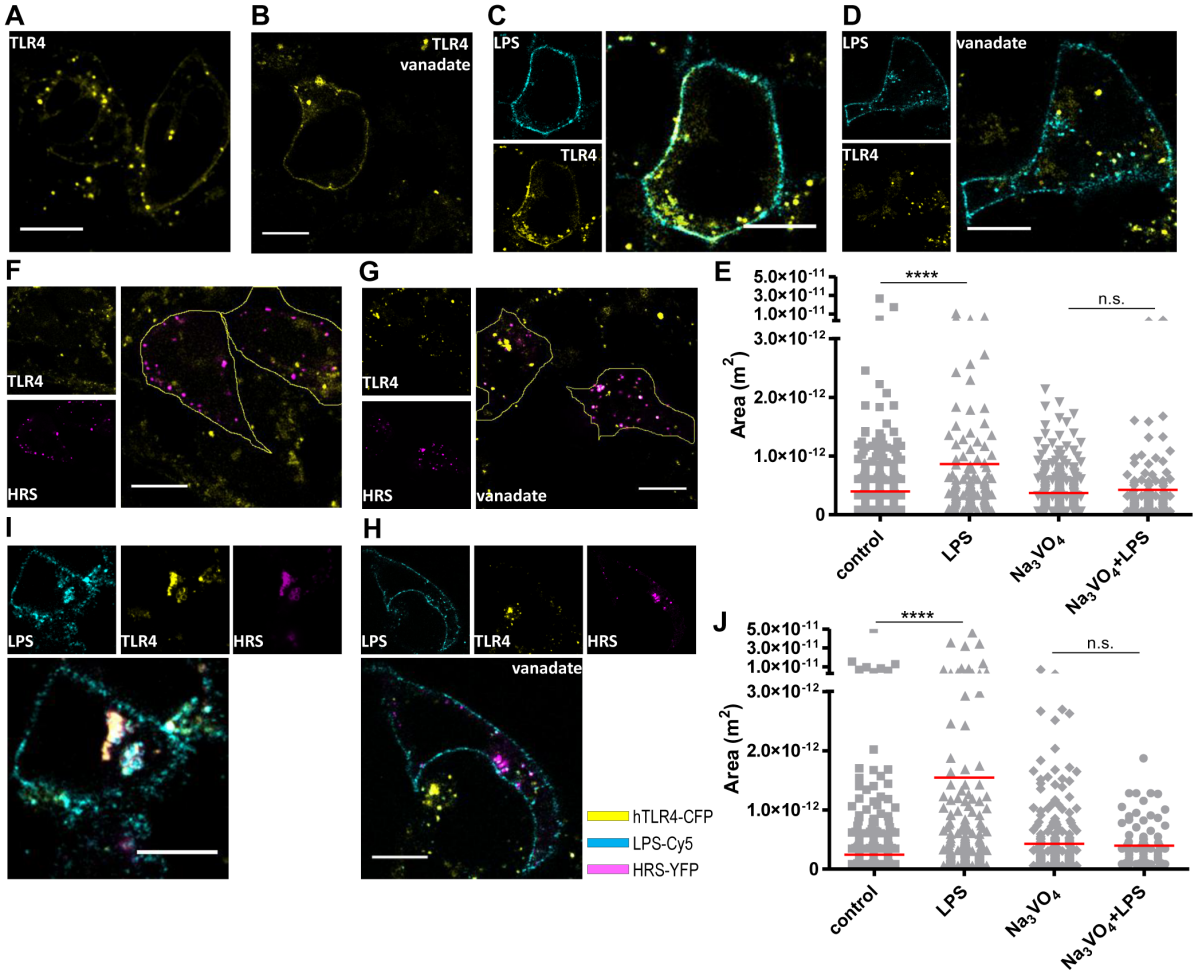
Vanadate-inhibited endosome fusion. HEK293 cells stably expressing TLR4-CFP/MD-2 were untreated (**A.**), stimulated with Na_3_VO_4_ (500 µM) (**B.**), LPS-Cy5 (2 µg/ml) (**C.**), or prestimulated for 10 min with Na_3_VO_4_ and stimulated for 1 h with LPS (**D.**). Cells were fixed and confocal imaging was performed. TLR4-positive endosome sizes were measured (**E.**). **F–J.** The same experiment was performed on HEK293 cells stably expressing TLR4-CFP/MD-2 and transfected with Hrs-YFP. Hrs/TLR4-positive endosome sizes were measured. Bar 20 µm. Representative examples of two (A–E and F–J) independent experiments are shown.

To show that vanadate indeed inhibits endosome fusion, we measured the size of endsomes in the presence and absence of vanadate pretreatment. HEK293 cells, stably transfected with TLR4-CFP/MD-2, were left untreated as a control, stimulated with LPS, with vanadate alone or with both (Figures 4A–D). TLR4-positive endosomes were measured in several images (Figure 4E). LPS stimulation increased the size of endosomes; on the other hand vanadate decreased endosome size to the level of the control. The cells transfected with Hrs-YFP were treated in the same way (Figures 4F–I). In untreated cells, Hrs and TLR4 colocalization was 39%. Stimulation with LPS increased colocalization to 66%. When Hrs was overexpressed, the endosomes were already larger without stimulation as well as in LPS stimulated cells (Figure 4J), while the vanadate decreased their size. Importantly, vanadate decreased the size of Hrs/TLR4-positive endosomes to the size of TLR4-positive endosomes observed in cells without Hrs overexpression. The results demonstrate that vanadate strongly inhibits endosome fusion, and thus influences LPS signaling, as shown in Figure 3C.

### Phosphorylation of Hrs is Important for Fusion Activity and TLR4 Degradation

**Figure 5:**
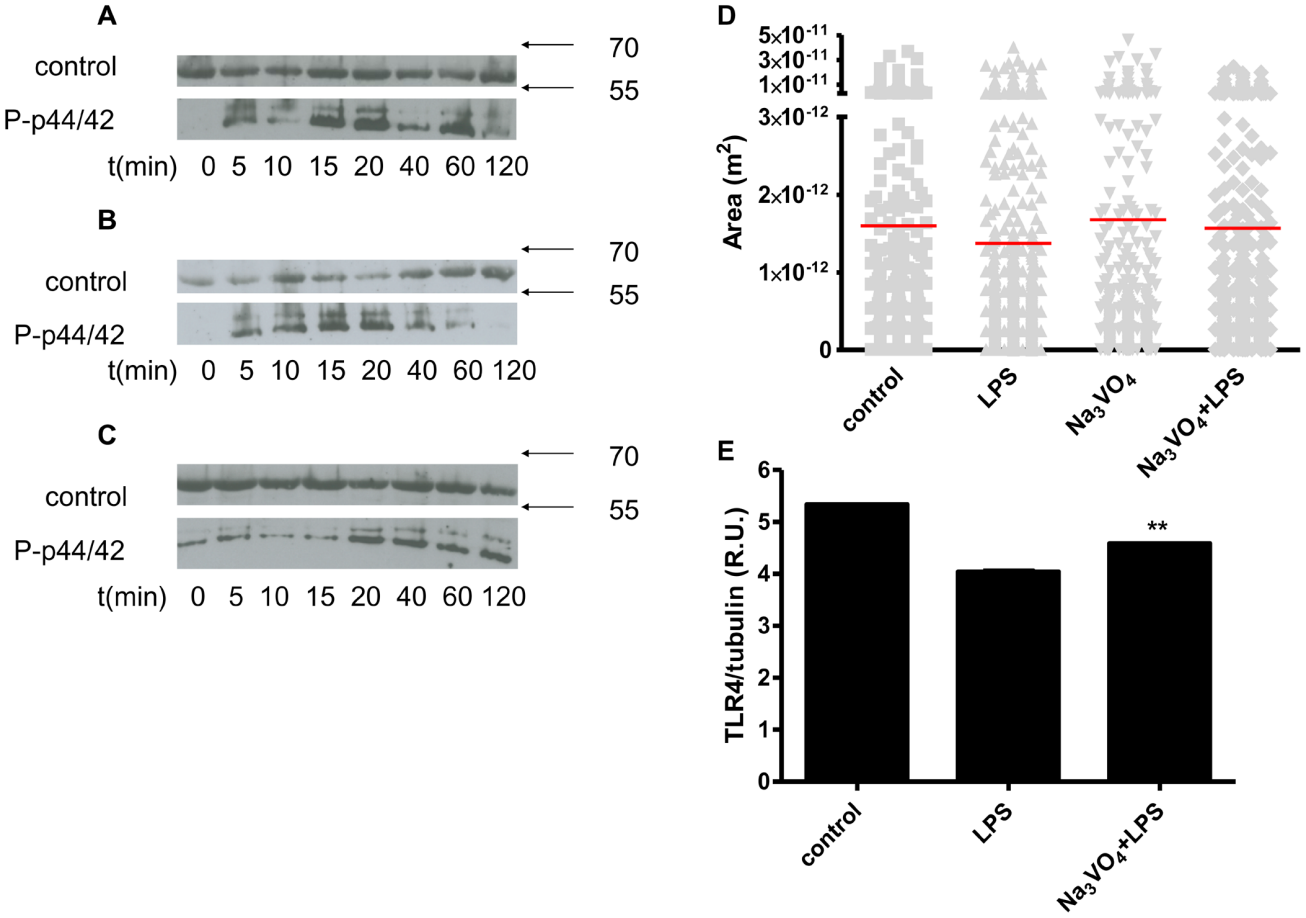
Vanadate increased kinase phosphorylation, inhibited TLR4 degradation after LPS stimulation and Hrs Y329/334F mutations may explain the process. RAW264.7 cells were stimulated in a timeframe between 0 to 120 minutes with Na_3_VO_4_ (2 mM) (**A.**), LPS (100 ng/ml) (**B.**) or both (**C.**). Cells were lysed, and the amount of phosphorylated p44/42 (P-p44/42) was detected. Anti-tubulin Abs were used as a control for equal protein loading. **D.** HEK293 cells stably expressing TLR4-CFP/MD-2 and transfected with HrsY329/334F-EGFP were prestimulated for 10 min with Na_3_VO_4_ (500 µM) and/or stimulated for 1 h with LPS-Cy5 (2 µg/ml). Cells were fixed and confocal imaging was performed. Hrs-positive endosome sizes were measured. The differences were not significant. **E.** HEK293 cells stably expressing TLR4-CFP/MD-2 and transfected with Hrs were pretreated with cycloheximide (80 µg/ml) for 30 minutes and prestimulated for 10 min with Na_3_VO_4_ (500 µM) and stimulated with LPS (100 ng/ml). After 3 h cells were lysed, 20 µg of proteins were loaded, and TLR4-CFP was detected on WB using anti-GFP Abs; the intensities of bands were normalized using anti-tubulin Abs. Representative examples of three (A–C and E) and one (D) independent experiments are shown.

In addition to ROS production, vanadate can also act as an inhibitor of tyrosine phosphatases, acting as a structural mimic of a phosphate group [26]. Treatment of macrophages with vanadate increased phosphorylation of MAPK p44/42, with two maximums at 15 and 60 min (Figure 5A). After LPS stimulation, phosphorylation of p44/42 peaked at 15 minutes and then declined (Figure 5B), but when the cells were stimulated with vanadate and LPS p44/42, phosphorylation remained at the maximum level for at least 100 minutes (Figure 5C). We reasoned this prolonged phosphorylation of kinases might influence the phosphorylation status of Hrs protein.

Hrs protein contains domains involved in sequence-directed recycling [27] and lysosomal-vacuolar sorting [28]. Outside of those domains, the protein comprises several potential phosphorylation sites on tyrosines (Y216, Y308, Y329 and Y334), where Y329 and Y334, specifically, have been recognized as being important for protein function [28], [29], [30]. We transfected cells stably expressing TLR4-CFP/MD-2 with Hrs containing Y329F and Y334F (HrsY329/334F-EGFP) mutations. Comparing the sizes of endosomes (Figure 5D) revealed neither vanadate nor LPS had any effect on the size of endosomes when Hrs double mutant was expressed, confirming that tyrosine phosphorylation is important for the fusion. Additionally, vanadate pretreatment resulted in decreased TLR4 degradation in comparison to LPS stimulation alone (Figure 5E), leading to a conclusion that vanadate causes increased Hrs phosphorylation, which in turn leads to the inhibition of endosome fusion and TLR4 degradation. Thus, as a result, LPS signaling is increased.

## Discussion

Among contaminants that are generated by fossil fuel combustion and released into the air, ROFA exhibits oxidative and inflammatory reactivity, primarily due to its high content of transition metals. Transition metals are adsorbed into ROFA, but are highly water soluble and capable of causing damage to biological macromolecules [9], [16]. Further, the water soluble fraction of ROFA has low vanadium and nickel ion content and high concentrations of iron [31]. Metal ions are released from inhaled ROFA into the fluid that surrounds lung epithelial cells and can cause the generation of reactive oxygen species (ROS) in cells [10], [32], [33], which causes injuries to the lung epithelia [34]. We showed that the addition of vanadate substantially increased responsiveness to bacterial ligands, which activate TLRs, and is in agreement with increased susceptibility to infections of persons exposed to ROFA [17].

ROS is an important mediator of stress, inflammation, and cell cycles, and also influences several other cell processes [35], [36] leading to inflammatory cytokine production, production of proteins, involved in cell proliferation and others. Vanadate in combination with IFNα even promotes ROS-dependent apoptosis in transformed cell lines [36]. Although we showed that vanadate pretreatment increased ROS production after LPS stimulation, preincubation with antioxidant NAC did not reduce the synergistic effect of vanadate on LPS-induced TLR4 signaling, implying that a different mechanism is more relevant.

Mucin induction by pathogens is phosphotyrosine dependent, and it had been suggested that vanadium-containing air pollutants trigger lung mucin production by unmasking phosphorylation-dependent pathogen resistant pathways [37]. Herein we have identified that vanadate inhibits endosome fusion by increased phosphorylation of the Hrs protein. Hrs is the main component in clathrin-coated endosomes, which specifically binds to the lipid phosphatidylinositol (PI) 3-phosphate by FYVE domain [38]. It is essential for sorting ubiquitinated proteins through the UIM (ubiquitin-interacting motif) domain into the lysosomal degradation pathway [20], [39]. The UIM domain is necessary for the EGF-stimulated (epidermal growth factor) tyrosine phosphorylation of Hrs [28]. Stern et al. proposed a mechanism where phosphorylation of Y329 and Y334 regulates Hrs degradation and showed that the level of Hrs phosphorylation and the speed of Hrs dephosphorylation correlated directly with EGFR degradation [29]. We observed decreased TLR4 degradation after vanadate pretreatment, suggesting a similar mechanism to Stern’s findings. The difference is probably in the way Hrs is phosphorylated. We observed smaller endosomes already in the presence of vanadate (Figures 3A or 4J), but NF-κB signaling and cytokine production were not affected by the vanadate alone. These results imply that Hrs phosphorylation is independent of LPS-mediated TLR4 stimulation, probably through Syk [30] and PI3-kinases [38], [40]. On the other hand, synergistic activity on NF-κB signaling depends on LPS signaling, probably through Syk and MAP kinase, as we have demonstrated.

To conclude, our results identify the mechanism of potentiating inflammation by air pollutant vanadate through the augmentation of the innate immune responses to infections and to endogenous danger signals, thus confirming their harmful effect on humans.
